# Serum Levels of Soluble Forms of Fas and FasL in Patients with Pancreatic and Papilla of Vater Adenocarcinomas

**DOI:** 10.3390/cancers18010106

**Published:** 2025-12-29

**Authors:** Stavros Anagnostoulis, Helen Bolanaki, Byron Asimakopoulos, Dimitrios Ouroumidis, Maria Koutini, Spyridon Patris, Ioannis Tzimagiorgis, Anastasios J. Karayiannakis

**Affiliations:** 1 Second Department of Surgery, Medical School, Democritus University of Thrace, 68100 Alexandroupolis, Greece; anagstav@hotmail.com (S.A.); bolanakie@gmail.com (H.B.); dimitrisourou@gmail.com (D.O.); koutinimaria@gmail.com (M.K.); patris.spyridon@gmail.com (S.P.); tzimagiorgis@hotmail.com (I.T.); 2 Laboratory of Physiology, Medical School, Democritus University of Thrace, 68100 Alexandroupolis, Greece; basima@med.duth.gr

**Keywords:** pancreatic carcinoma, Vater carcinoma, sFas, sFasL, surgery, prognosis, survival

## Abstract

**Objective:** Impairment of the Fas/FasL apoptotic pathway is a mechanism contributing to the malignant transformation of multiple cell types. This study aimed to investigate the clinical and prognostic relevance of serum soluble Fas (sFas) and soluble Fas ligand (sFasL) levels in patients with pancreatic and papilla of Vater adenocarcinomas. **Methods:** An ELISA was used to determine sFas and sFasL levels. Serum samples were obtained from 53 healthy controls, 82 pancreatic and 14 papilla of Vater carcinoma patients. Sera from carcinoma patients were obtained before surgery and 30 days after surgery. The relationships of preoperative levels with clinicopathological features and patient survival were evaluated. Changes in serum sFas and sFasL levels after surgery were also evaluated. **Results:** Higher sFas and lower sFasL levels were found in the serum of carcinoma patients in comparison to healthy controls. Serum sFas and sFasL levels correlated significantly with both lymph node and distant metastases and an advanced stage of disease. Elevated sFas and decreased sFasL levels correlated significantly with poor overall survival when the entire study population was considered with sFas being an independent prognostic factor. After patient stratification, their prognostic value was evident in pancreatic carcinoma patients only. Preoperative sFas levels decreased and sFasL levels increased after radical resection of the tumor but remained unchanged in cases of unresectable disease. **Conclusions:** These findings suggest that serum levels of sFas and sFasL could be useful tumor markers with prognostic value in pancreatic adenocarcinomas. Increased sFas secretion may reflect a mechanism for apoptotic escape of cancer cells.

## Simple Summary

Impairment of the Fas/FasL apoptotic pathway is a mechanism contributing to malignant transformation and may facilitate immune escape by reducing apoptosis. We evaluated the serum levels of soluble forms of Fas receptor (sFas) and its ligand (sFasL) in patients with pancreatic and papilla of Vater adenocarcinomas as well their changes after surgery. The results showed higher sFas and lower sFasL levels in cancer patients compared to healthy controls. These levels correlated with lymph node and distant metastases and advanced disease stage, as well as their changes associated with resectional surgery. Altered levels of sFas and sFasL and sFas/sFasL ratio correlated with poor overall survival of pancreatic carcinoma patients. These findings suggest that serum levels of sFas and sFasL could be useful tumor markers in pancreatic or papilla of Vater adenocarcinomas and may reflect a mechanism of escape of cancer cells from apoptosis induction.

## 1. Introduction

The Fas, also known as Apo-1 or CD95, is a 45 kDa type I transmembrane protein that belongs to the nerve growth factor–tumor necrosis factor receptor family [1], whereas its natural ligand, Fas ligand (FasL), is a 40 kDa type II membranous protein belonging to the tumor necrosis factor (TNF) ligand superfamily [2]. FasL binding to Fas induces apoptotic death of cells expressing functional Fas, with Fas/FasL-mediated apoptosis being an important pathway in the regulation of immune response and tissue homeostasis [3,4,5].

Fas is constitutively expressed in most human tissues and in lymphocytes [5,6], whereas FasL is expressed abundantly in activated T lymphocytes and natural killer cells and in immunoprivileged tissues such as the placenta [7], the testis and the anterior chamber of the eye [8] where FasL-expressing cells induce apoptosis in Fas-expressing infiltrating lymphocytes [6,7]. Apart from their membrane bound form, both Fas and FasL also exist in a soluble form. Soluble Fas (sFas) is generated by alternative mRNA splicing [9], whereas soluble FasL (sFaL) is produced by metalloprotease cleavage of the membrane bound protein [10,11].

Alterations in the expression of both Fas and FasL have been documented in hemopoietic and solid malignancies including pancreatic cancer [12,13,14,15] and may represent a mechanism by which cancer cells escape immune surveillance [16,17,18]. FasL-expressing tumor cells counterattack the host immune system by inducing apoptosis in Fas-expressing antitumor T lymphocytes. On the other hand, loss of Fas expression and/or function allows cancer cells to escape the physiological Fas-mediated apoptotic pathways resulting in tumor progression and metastasis as has been shown in experimental studies [19,20].

Elevated levels of sFas have been detected in the serum of patients with hemopoietic malignancies and solid tumors in a manner directly related to tumor stage and burden [21,22,23]. Tumor-derived sFas may suppress tumor cell apoptosis by blocking FasL on lymphocytes, thus facilitating tumor progression [24,25]. The proteolytically cleaved sFasL has been shown to be an inducer of apoptosis, although it is less active than the membrane-bound FasL [26]; therefore, elevated serum levels of sFasL in cancer patients [27,28,29] may represent an additional mechanism of immune escape [30,31]. These findings underline the complex interactions between Fas and FasL within the Fas/FasL system and suggest more complex roles for these molecules at the interface of tumor cells with the host immune system. Therefore, simultaneous evaluation of both Fas and FasL levels is very important when studying their expression and/or function in cancer patients. The clinical significance of both sFas and sFasL in patients with pancreatic and papilla of Vater carcinomas has not been studied before, and their prognostic value is unknown.

Pancreatic head and papilla of Vater adenocarcinomas have similar clinical presentation and are largely treated by the same type of surgery. However, they are distinct entities with different biology and prognosis. We assumed that simultaneous measurement of sFas/sFasL and calculation of their ratio would reflect better their changes during the progression of these two types of adenocarcinomas. In addition, changes in sFas/sFasL levels and in their ratio according to the type of treatment may reveal the dynamics of the sFas/sFasL system.

In this study, we evaluated the serum concentrations of sFas and sFasL and their ratio in patients with pancreatic and papilla of Vater carcinomas in comparison with healthy controls and assessed their relationship to clinicopathological features and patient survival. The changes in preoperative sFas and sFasL levels after tumor surgery were also evaluated.

## 2. Patients and Methods

### 2.1. Patients and Healthy Controls

One hundred twenty-three consecutive patients with a presumptive diagnosis of pancreatic or papilla of Vater adenocarcinoma were studied prospectively. In 27 patients, the final diagnosis was benign disease or tumors of the periampullary area other than pancreatic or papilla of Vater adenocarcinomas. These patients were excluded from further analysis. The remaining 96 patients (67 men and 29 women; mean age 70 ± 9 (range, 43–93) years) had pancreatic ductal adenocarcinomas (82 patients) or adenocarcinoma of the papilla of Vater (14 patients) and were included in the study. No patient had received chemotherapy, irradiation or any intervention for the relief of jaundice before blood sampling. The presence of any inflammatory conditions was excluded by clinical history and physical examination and detailed laboratory testing. Tumor staging was based on radiological reports (abdominal ultrasonography, computerized tomography and magnetic resonance imaging), operative findings and pathology reports and was made according to the 6th edition of the TNM classification of the International Union Against Cancer available at the study commencement (2005). Patients were followed prospectively and the dates and causes of death recorded.

Fifty-three volunteers (35 men and 18 women) with a mean age of 67 ± 11 years (range 42–82 years) served as a control group. Their healthy condition was confirmed by clinical history and physical examination along with complete blood count, liver and renal function tests in order to exclude anemia, infections and inflammation.

The study was conducted in accordance with the Declaration of Helsinki and approved by the appropriate institutional authority. Informed consent was obtained from control subjects and carcinoma patients.

### 2.2. Blood Samples and Assays

Blood samples were collected from a peripheral vein in the morning (8.00–9.00 h) after an overnight fast. Blood was collected into sterile glass tubes (Vacutainer, Becton Dickinson, Plymouth, UK) and allowed to coagulate at room temperature for 30 min. Serum was separated by centrifugation at 2000× *g* for 10 min, aliquoted and stored at −80 °C until analyzed. Serum samples from carcinoma patients were obtained on admission and 30 days after treatment according to our standard protocol for all cancer patients. Clinical examination and detailed laboratory testing were performed at the same time points, and blood samples were obtained for measurement of Fas and FasL.

Serum sFas and sFasL concentrations were determined using solid-phase, enzyme-linked immunosorbent assays (ELISAs) designed to measure soluble levels of Fas and FasL in cell culture supernatant, serum and plasma (Quantikine, R&D Systems, Minneapolis, MN, USA). Both assays employ the quantitative sandwich enzyme immunoassay technique using recombinant human Fas and FasL with antibodies raised against the recombinant proteins, respectively. Their sensitivity was 20 pg/mL for sFas and 1 pg/mL for sFasL. Intra-assay and inter-assay coefficients of variation were 2.9–4.6% and 2.9–6.7% for sFas and 3.1–3.6% and 3.6–4.5% for sFasL, respectively. The log/log curve-fit mode was used to generate the standard curves for each set of samples assayed. Optical density was measured at 450 nm using a microtiter plate reader (Anthos 2001, Dynatech Laboratories, Chantilly, VA, USA) with the wavelength correction set to 570 nm. The serum concentrations of sFas and sFasL were extracted from the standard curves, and their ratio was calculated. An investigator who was blinded to the diagnosis and clinical details performed all measurements in duplicate.

### 2.3. Statistical Analysis

The distributions of the data were tested by the Kolmogorov–Smirnov test and were found to be normally distributed. Therefore, the results are presented as mean values ± standard deviation (SD), with parametric tests being employed to assess statistically significant differences. Differences between multiple groups were evaluated by analysis of variance (ANOVA) followed by *t*-test with Bonferroni correction for multiple comparisons. Two-tailed unpaired and paired *t*-tests were used when appropriate. The Cox proportional hazards regression model was used for the multivariate analysis after univariate analysis had defined relevant prognostic variables. Survival curves were obtained by the Kaplan–Meier method, and comparisons were made with the log-rank test. Significance was presumed at *p* < 0.05.

## 3. Results

### 3.1. Serum Levels of sFas, sFasL and sFas/sFasL Ratio in Carcinoma Patients and Healthy Controls

**Figure 1:**
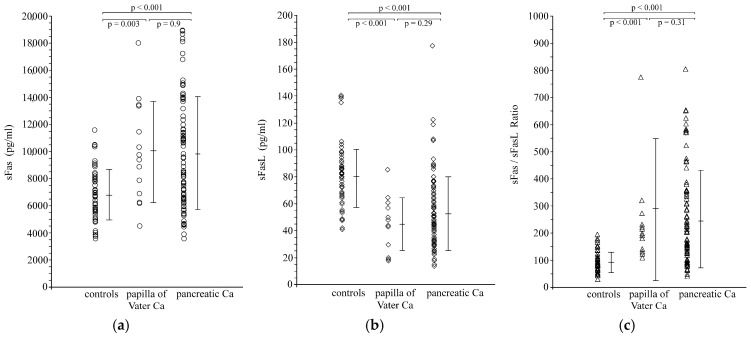
Scattergrams of serum levels of (**a**) sFas; (**b**) sFasL; and (**c**) sFas/sFasL ratio in control subjects and in carcinoma patients.

Serum levels of sFas and sFasL in control subjects were 6784 ± 1864 pg/mL and 80.6 ± 23.2 pg/mL, respectively. Their sFas/sFasL ratio was 91.2 ± 37.5. Pancreatic and papilla of Vater carcinoma patients as a group showed significantly higher preoperative sFas levels (9871 ± 4078 pg/mL; *p* < 0.001), significantly lower sFasL levels (51.7 ± 26.2 pg/mL; *p* < 0.001) and a significantly higher sFas/sFasL ratio (251.9 ± 188.6; *p* < 0.001) when compared with healthy controls (Table S1). These findings remained valid also when pancreatic and papilla of Vater carcinoma patients were considered as separate groups and compared with the healthy controls (Figure 1).

There were no statistically significant differences regarding sFas and sFasL levels and their ratio among patients with pancreatic or papilla of Vater adenocarcinoma (Table 1).

### 3.2. Correlations Between Serum Levels of sFas, sFasL and sFas/sFasL Ratio in Carcinoma Patients and Pathological Features

Patients with lymph node metastases had significantly (*p* = 0.04) higher sFas levels, significantly (*p* = 0.03) lower sFasL levels and a significantly (*p* = 0.04) higher sFas/sFasL ratio when compared with those without lymph node involvement. The patients with distant metastases also had significantly (*p* < 0.001) higher sFas levels, significantly (*p* = 0.03) lower sFasL levels and a significantly (*p* < 0.001) higher sFas/sFasL ratio in comparison with patients without distant metastases. The levels of sFas correlated significantly (*p* < 0.001, ANOVA) with disease stage, with higher sFas levels detected as the disease stage increased. The levels of sFasL also correlated significantly (*p* = 0.04, ANOVA) with disease stage, with lower sFasL levels related to an increased stage of disease. Finally, the sFas/sFasL ratio correlated significantly (*p* < 0.001, ANOVA) with disease stage with higher ratios reflecting advanced disease stage (Table 2).

There were no significant relationships between sFas and sFasL levels and their ratio with tumor size (T class) and degree of differentiation.

### 3.3. Correlations Between Serum Levels of sFas, sFasL and sFas/sFasL Ratio and Patient Survival

Since data were normally distributed, the normal ranges for sFas, sFasL and sFas/sFasL ratio were defined as the mean ± 2SD of the respective values in the healthy control group. The resulted ranges were 3055–10,513 pg/mL for sFas, 34–127 pg/mL for sFasL and 16–166 for their ratio. Using these cut-off limits, abnormally elevated sFas levels were found in 42/96 patients (43.7%): 37/82 (45.1%) and 5/14 (35.7%) in pancreatic and papilla of Vater adenocarcinoma, respectively. Abnormally low sFasL levels were found in 25/96 patients (26%): 21/82 (25.6%) and 4/14 (28.6%) in pancreatic and papilla of Vater adenocarcinoma, respectively. In contrast, 53/96 patients (55.2%): 43/82 (52.4%) and 10/14 (71.4%) in pancreatic and papilla of Vater adenocarcinoma, respectively, showed an abnormally elevated sFas/sFasL ratio.

**Figure 2:**
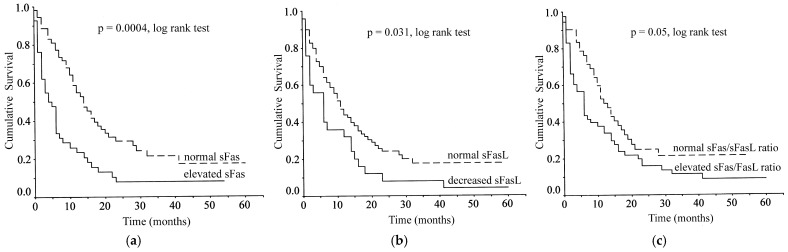
Kaplan–Meier survival curves according to serum levels of (**a**) sFas; (**b**) sFasL; and (**c**) sFas/sFasL ratio in carcinoma patients.

In this series, there were four deaths during the first postoperative month because of postoperative complications. These patients were also included in survival analysis as censored cases. Patient follow-up ranged between 17 and 62 months (mean 40.4 ± 12.8 months) with one papilla of Vater carcinoma patient being lost to follow-up. During this period, 75 patients died from disease progression and 16 patients remained alive (10 pancreatic and 6 papilla of Vater carcinoma patients). Log-rank analysis showed elevated sFas levels (*p* = 0.0004), decreased sFasL levels (*p* = 0.031) and elevated sFas/sFasL ratio (*p* = 0.05) to correlate with poor overall survival (Figure 2).

The mean survival of 42 patients with elevated sFas levels (>10,513 pg/mL) was significantly lower than that of 53 patients with nonelevated levels (9.77 months, 95% confidence interval (CI): 5.41–14.14 vs. 21.59 months, 95% CI: 16.01–27.18, log-rank test, χ^2^ = 12.75, *p* = 0.0004). Four patients with elevated sFas and 12 patients with normal sFas remained alive during the follow-up.

The mean survival of 25 patients with decreased sFasL levels (<34 pg/mL) was significantly lower than that of 70 patients with normal levels (10.40 months, 95% CI: 5.04–15.76 vs. 18.84 months, 95% CI: 13.95–23.73, log-rank test, χ^2^ = 4.65, *p* = 0.031). Fifteen patients with normal sFasL and one patient with abnormally low sFasL remained alive during the follow-up.

The mean survival of 53 patients with elevated sFas/sFasL ratio (>166) was significantly lower than that of 42 patients with normal ratio (13.35 months, 95% CI: 8.64–18.06 vs. 19.96 months, 95% CI: 14.14–25.79, log-rank test, χ^2^ = 3.84, *p* = 0.05). Six patients with elevated sFas/sFasL ratio and 10 patients with normal ratio remained alive during the follow-up.

However, tumor-type-adjusted survival analysis revealed that the statistical significances in the pooled data were achieved because of the pancreatic carcinoma patients. Significant survival correlations were found in pancreatic carcinoma for sFas (log-rank test, χ^2^ = 11.71, *p* = 0.0006), sFasL (log-rank test, χ^2^ = 6.01, *p* = 0.014) and sFas/sFasL ratio (log-rank test, χ^2^ = 5.23, *p* = 0.022). In contrast, there were no significant correlations in papilla of Vater carcinoma for sFas (log-rank test, χ^2^ = 2.5, *p* = 0.113), sFasL (log-rank test, χ^2^ = 0.82, *p* = 0.366) and sFas/sFasL ratio (log-rank test, χ^2^ = 3.25, *p* = 0.071). These results are probably related to the small number of cases and low overall death rate in the papilla of Vater carcinoma group.

Univariate analysis showed tumor type, tumor resection, tumor size, lymph node metastasis, distant metastasis, TNM stage and sFas and sFasL levels to be significant factors affecting overall survival. The prognostic value of the sFas/sFasL ratio was found to be marginally significant (Table 3). Multivariate regression analysis revealed that the type of the tumor (hazard ratio (HR): 3.04; 95% CI: 1.27–7.27; *p* = 0.012), tumor resection (HR: 1.71; 95% CI: 0.94–3.11; *p* = 0.077), sFas levels (HR: 1.92; 95% CI: 1.15–3.22; *p* = 0.013) and disease stage (HR: 1.5; 95% CI: 1.08–2.08; *p* = 0.014) are independent prognostic factors for patient survival.

### 3.4. The Effect of Surgery on Serum Levels of sFas, sFasL and sFas/sFasL Ratio

We assumed that possible changes in sFas and sFasL levels and in their ratio according to the type of treatment may expose the dynamics of the sFas/sFasL system. Radical resection for cure of the primary tumor was performed in 39 patients, whereas 57 patients underwent either palliative bypass surgery (52 patients) or endoscopic stenting (5 patients). To avoid any interference on the serum levels of Fas and FasL, four patients in each group (four patients who died in the postoperative period and another four patients with septic postoperative complications) were excluded from the analysis.

One month following radical tumor resection, there was a significant decrease in the serum levels of sFas (*p* = 0.026) and a significant increase in the serum levels of sFasL (*p* = 0.0001) with their values being not statistically different from those in the healthy control group. The sFas/sFasL ratio also decreased significantly (*p* = 0.002) following radical resection of the tumor when compared with preoperative values (*p* = 0.0002) but remained significantly higher than in control subjects (Table 4).

In contrast, postoperative serum levels of sFas, sFasL and their ratio in patients undergoing palliative bypass or endoscopic stenting did not differ from their corresponding values before intervention.

## 4. Discussion

In this study, both pancreatic and papilla of Vater carcinoma patients showed significantly higher serum levels of sFas, lower sFasL levels and higher sFas/sFasL ratio, when compared with healthy controls. Serum sFas and sFasL levels were associated with advanced disease and poor overall survival, but only sFas levels were found to be an independent prognostic factor in pancreatic carcinoma patients.

Pancreatic adenocarcinoma is one of the most aggressive tumors with poor response to therapy and dismal prognosis. Alterations in cell apoptosis such as diminished Fas/FasL-induced apoptosis may contribute to tumor progression and resistance to therapy [17,30,31,32]. Although multiple mechanisms are involved in the escape from host immune surveillance and apoptotic death of transformed cells, the Fas/FasL pathway plays a pivotal role in apoptosis. Our findings of aberrant serum levels of the soluble forms of Fas and FasL in cancer patients suggest a possible function in the loss of sensitivity of cancer cells to apoptosis. Pancreatic cells express both Fas and FasL, and loss of Fas has been correlated with malignant transformation and biologic aggressiveness in pancreatic adenocarcinoma [31]. Pancreatic cells have shown resistance to Fas-mediated apoptosis, a mechanism that possibly contributes to their malignant transformation. This is not due to receptor downregulation or Fas-associated phosphatase upregulation [16,33]. Interestingly, endogenous decoy receptor 3, a soluble receptor against FasL, has been correlated to FasL loss of sensitivity of pancreatic adenocarcinoma by antagonistically blocking the growth inhibition signals [26,34]. It has been suggested that sFas could provide apoptosis resistance through an autocrine manner with tumor-derived sFasL exerting a paracrine pro-apoptotic effect in the microenvironment of pancreatic cancers [25].

Our results are in accordance to those reported by Bellone et al. [25], showing significantly higher sFas levels in the serum of pancreatic cancer patients when compared to healthy controls. In our study, sFas levels were examined in both pancreatic and papilla of Vater carcinoma patients and were found elevated in both groups. In contrast, sFasL levels were found to be significantly lower in carcinoma patients. These results are in agreement to previous findings showing detectable serum levels of sFasL in only few patients with metastatic disease despite elevated sFasL secretion by pancreatic cancer cells [30,35]. Very low or no measurable levels of sFasL have been reported in prostate cancer patients [36]. Hepatocellular and bile duct carcinoma patients have also decreased serum sFasL levels compared to healthy subjects [37,38]. We also calculated the sFas/sFasL ratio assuming it as a better indicator of their dynamics. We found significantly higher ratios in carcinoma patients compared to healthy controls. This finding suggests a relative predominance of sFas. Based on our findings, it could be postulated that high levels of sFas secreted by tumor cells bind and neutralize a considerable fraction of sFasL. As a result, lower sFasL levels are detected in the serum.

Correlations of serum levels of sFas, sFasL and their ratio with features of tumor aggressiveness like lymph node and distant metastases and advanced stage were found. Furthermore, elevated sFas, decreased sFasL levels and elevated sFas/sFasL ratio correlated significantly with poor overall survival when the entire study population was considered with sFas being an independent prognostic factor. However, after patient stratification their prognostic value was evident only in pancreatic carcinoma but not in papilla of Vater carcinoma. The small numbers of patients and events in the papilla of Vater adenocarcinoma group probably have affected our results. Despite their similarity in clinical presentation and surgical approach, pancreatic head and papilla of Vater adenocarcinomas are distinct entities with different biology and prognosis. Their distinction is not always obvious when initially considered. In this context, we believe that our data pooling is reasonable. Increased serum sFas levels have been reported in a variety of human malignancies including gynaecological malignancies, melanoma, hepatocellular, bladder and renal cell carcinomas [23,28,39,40,41,42,43]. Serum sFas levels have been correlated with decreased overall survival in bladder cancer or metastatic prostate cancer and renal cell carcinoma [23,28,36,41,43]. Shortened disease-free and overall survival in ovarian cancer has been also reported [40]. Although aberrant Fas and FasL expression has been shown in pancreatic cancer, their prognostic value remains uncertain [14,15,17], and there is no evidence on their soluble forms as prognostic factors. Our finding of sFas being an independent prognostic factor for overall survival in pancreatic carcinoma patients suggests a prognostic potential. We also evaluated the sFas/sFasL ratio, suggesting that it may reflect better the alterations in the sFas/sFasL system. Elevated sFas/sFasL ratio correlated with poor overall survival in pancreatic carcinoma patients but failed to achieve an independent significance.

In this study, for the first time, we studied changes in sFas and sFasL levels according to the type of treatment assuming that this may depict the dynamics of the sFas/sFasL system. Serum levels of sFas decreased and of sFasL increased significantly, and their ratio was accordingly affected after resection for cure. Such changes were not observed in cases of palliative treatment. These findings suggest that elevated sFas levels in cancer patients associate with the tumor burden but do not establish causality. The sFas levels decreased after radical resection and did not differ from that of healthy subjects. These findings support the notion that the tumor is probably the source of Fas secretion. There is in vivo evidence that pancreatic tumors secrete sFas in the serum as opposed to the absence of sFas in the culture media of pancreatic carcinoma cell lines [25]. In contrast, serum sFasL levels increased after surgery, becoming not significantly different to that of control subjects. This was probably due to the absence of high postoperative serum concentration of sFas that would neutralize FasL. The sFas/sFasL ratio also decreased significantly but remained significantly higher than in control subjects. Whether these changes may serve as a possible indicator for potentially curative tumor resection remains to be documented. Further studies are needed to evaluate sFas and sFasL as potential monitoring biomarkers during follow-up.

Apoptosis is the physiological process of eliminating harmed cells, and its suppression is an essential step in carcinogenesis and in the development of resistance to chemotherapy and radiotherapy [17,30,31]. Understanding the molecular mechanisms involved could be crucial for patient prognosis, but also in designing new therapeutic anti-cancer strategies, aiming to restore the apoptotic regulation of tumor cells. Fas-mediated apoptosis is one of the major induction pathways and is often impaired in cancer cells, thus allowing their irregular growth. Nevertheless, the exact relationship between membrane and soluble forms of Fas and FasL has not been fully elucidated. Multiple mechanisms could be involved, including loss of the cell-surface Fas, rendering carcinoma cells resistant to apoptosis or receptor activation by a truncated ligand, such as the soluble form of FasL. Alternatively, neutralization of FasL by sFas would prevent the ligand from triggering apoptosis. There is some evidence that sFas suppresses Fas/FasL-mediated apoptosis, whereas sFasL induces apoptosis by binding to Fas expressing target cells [10,24,29]. Our findings of elevated sFas and decreased sFasL levels in carcinoma patients are in agreement with these observations. It is conceivable that increased sFas levels may correspond to suppressed apoptosis, and decreased sFasL levels may reflect diminished apoptosis in pancreatic and papilla of Vater carcinoma patients.

This study has several limitations. This is a single-center study, and the number of patients with papilla of Vater adenocarcinomas was very limited. In addition, our findings are lacking functional mechanistic validation. We demonstrated a prognostic value for sFas and sFasL and showed that their postoperative changes associate with radical resection. However, these results should be considered as preliminary and interpreted with caution. Further confirmation from different centers with a large number of cases is required to examine whether sFas and sFasL levels could be useful monitoring biomarkers postoperatively particularly when cancer-specific and disease-free survival are considered.

## 5. Conclusions

In conclusion, alterations in sFas and sFasL levels and in their ratio are found in pancreatic and papilla of Vater carcinoma patients and correlate with aggressive tumor characteristics. Neutralization of FasL by increased concentrations of sFas may represent a mechanism for immune escape of cancer cells. Postoperative shifts according to the type of treatment could reveal the dynamics of the sFas/sFasL system. Serum sFas level is an independent prognostic factor in pancreatic adenocarcinomas that is potentially of clinical usefulness.

## Figures and Tables

**Table 1:** Serum levels of sFas, sFasL and their ratio in healthy controls and in patients with pancreatic and papilla of Vater adenocarcinomas.

	Controls (*n* = 53)	Pancreatic Carcinoma (*n* = 82)	Papilla of Vater Carcinoma (*n* = 14)
Age (years)	67 ± 11	70 ± 9	68 ± 10
Sex (M/F)	35/18	54/28	13/1
sFas (pg/mL)	6784 ± 1864	9852 ± 4158 *	9980 ± 3716 *
sFasL(pg/mL)	80.6 ± 23.2	52.8 ± 27.2 *	45.1 ± 19.2 *
sFas/sFasL ratio	91.2 ± 37.5	245.4 ± 175.3 *	290.4 ± 258.2 *

Values are mean ± SD. * Statistically significant differences patients vs. controls (*p* < 0.001).

**Table 2:** Relationship between serum levels of sFas, sFasL and their ratio and pathological variables in patients with pancreatic and papilla of Vater carcinomas.

	sFas (pg/mL)	sFasL(pg/mL)	sFas/sFasLRatio
Tumor class			
T_1_ (*n* = 4)	10,312 ± 3453	60.6 ± 24.3	216.3 ± 170.9
T_2_ (*n* = 14)	10,261 ± 4462	59.2 ± 29.3	235.7 ± 194.2
T_3_ (*n* = 53)	9179 ± 3565	51.4 ± 27.5	237.9 ± 176.8
T_4_ (*n* = 25)	11,049 ± 4836	46.5 ± 21.8	296.5 ± 214.8
Lymph node metastases			
N_0_ (*n* = 30)	8750 ± 3222	59.8 ± 32.3	198.2 ± 156.2
N_1_ (*n* = 66)	10,380 ± 4340 *	47.9 ± 22.3 *	276.4 ± 197.9 *
Distant metastases			
M_0_ (*n* = 75)	8632 ± 3317	54.3 ± 27.2	211.8 ± 175.3
M_1_ (*n* = 21)	14,296 ± 3458 **	42.2 ± 20.2 **	395.3 ± 166.3 **
TNM stage			
I (*n* = 10)	8450 ± 2564	67.9 ± 24.7	139.9 ± 62.8
II (*n* = 50)	8507 ± 3217	53.6 ± 27.8	212.5 ± 169.6
III (*n* = 15)	9170 ± 4163	47.8 ± 25.3	257.5 ± 230.9
IV (*n* = 21)	14,296 ± 3458 ***	42.2 ± 20.2 ***	395.3 ± 166.3 ***
Tumor differentiation			
Well (*n* = 10)	8322 ± 2874	55.5 ± 28.6	196.3 ± 147.1
Moderate (*n* = 22)	9628 ± 3658	50.3 ± 33.1	266.5 ± 199.7
Poor (*n* = 15)	8796 ± 3624	53.5 ± 22.9	200.1 ± 114.1

Values are mean ± SD. Statistically significant differences; * N_0_ vs. N_1_ (*t*-test); ** M_0_ vs. M_1_ (*t*-test); *** TNM stage (ANOVA).

**Table 3:** Univariate analysis for predictors of overall patient survival.

Variable	HR	95% CI	*p*-Value
Age	1.01	0.98–1.03	0.45
Sex (Male vs. Female)	0.76	0.47–1.24	0.27
Tumor type (Pancreas vs. Papilla of Vater)	3.7	1.58–8.67	0.003
Tumor resection (curative vs. unresectable)	2.60	1.59–4.25	0.000
Differentiation (Well vs. Moderate vs. Poor)	1.49	0.88–2.53	0.137
T Class (T_1_ vs. T_2_ vs. T_3_ vs. T_4_)	1.89	1.34–2.67	0.000
Nodal status (N_0_ vs. N_1_)	1.98	1.19–3.29	0.008
Distant metastases (M_0_ vs. M_1_)	3.58	1.99–6.44	0.000
TNM Stage (I vs. II vs. III vs. IV)	2.05	1.57–2.68	0.000
sFas (Elevated vs. Normal)	2.12	1.33–3.36	0.001
sFasL (Elevated vs. Normal)	1.72	1.05–2.81	0.032
sFas/FasL ratio (Elevated vs. Normal)	1.51	0.95–2.40	0.078

HR, hazard ratio; CI, confidence interval.

**Table 4:** Changes in serum sFas and sFasL concentrations and their ratio according to type of surgery.

	Controls	Before Surgery	After Surgery
Radical	*n* = 53	*n* = 39	*n* = 35
sFas (pg/mL)	6784 ± 1864	8584 ± 2975 *	7231 ± 2755 **
sFasL(pg/mL)	80.6 ± 23.2	54.5 ± 30.4 *	72.9 ± 37.3 **
sFas/sFasL ratio	91.2 ± 37.5 ***	212.3 ± 158.9 *	119.7 ± 72.3 **
Palliative/unresectable	*n* = 53	*n* = 57	*n* = 53
sFas (pg/mL)	6784 ± 1864 ***	10,752 ± 4502 *	11,016 ± 4258
sFasL(pg/mL)	80.6 ± 23.2 ***	49.7 ± 23.1 *	51.2 ± 18
sFas/sFasL ratio	91.2 ± 37.5 ***	279.1 ± 203.4 *	256.2 ± 165.7

Values are mean ± SD. Statistically significant differences; * preoperative vs. controls; ** postoperative vs. preoperative; *** controls vs. postoperative.

## Data Availability

The data presented in this study are available on request from the corresponding author.

## Supplementary Materials

The following supporting information can be downloaded at: https://www.mdpi.com/article/10.3390/cancers18010106/s1, Table S1: Raw data on sFas and sFasL measurements.
